# Association Between Sociodemographic and Lifestyle Factors and Type 2 Diabetes Risk Scores in a Large Working Population: A Comparative Study Between the Commerce and Industry Sectors

**DOI:** 10.3390/nu17152420

**Published:** 2025-07-24

**Authors:** María Pilar Fernández-Figares Vicioso, Pere Riutord Sbert, José Ignacio Ramírez-Manent, Ángel Arturo López-González, José Luis del Barrio Fernández, María Teófila Vicente Herrero

**Affiliations:** 1 Obesity and Metabolic Syndrome Group, Spanish Association of Specialists in Occupational Medicine, 28012 Madrid, Spain; pfigares@gmail.com (M.P.F.-F.V.); correoteo@gmail.com (M.T.V.H.); 2 ADEMA-Health Group of IUNICS, University of Balearic Islands, 07122 Palma, Spain; pereriutord@gmail.com (P.R.S.); jignacioramirez@telefonica.net (J.I.R.-M.); 3 Health Research Institute of the Balearic Islands (IDISBA), 07120 Palma, Spain; 4 Faculty of Medicine, University of Balearic Islands, 07122 Palma, Spain; 5 Faculty of Health Sciences, Rey Juan Carlos University, 28032 Madrid, Spain; jose.delbarrio@urjc.es

**Keywords:** type 2 diabetes, lifestyle, Finrisk, physical activity, mediterranean diet, T2D risk scores

## Abstract

Background: Type 2 diabetes (T2D) is a major global health concern influenced by sociodemographic and lifestyle factors. This study compared T2D risk scores between commerce and industry sectors and assessed the associations of age, sex, education, physical activity, diet, and smoking with elevated risk. Methods: This cross-sectional study included 56,856 men and 12,872 women employed in the commerce (*n* = 27,448) and industry (*n* = 42,280) sectors across Spain. Anthropometric, clinical, and biochemical data were collected. Four validated T2D risk scores (QDscore, Finrisk, Canrisk, and TRAQ-D) were calculated. Multinomial logistic regression models estimated adjusted odds ratios (ORs) for high-risk categories by sociodemographic and lifestyle characteristics. Results: Women in the industrial sector had significantly higher age, BMI, waist circumference, and lipid levels than those in commerce; differences among men were less marked. Across all participants, higher T2D risk scores were independently associated with physical inactivity (OR up to 12.49), poor Mediterranean diet adherence (OR up to 6.62), industrial employment (OR up to 1.98), and older age. Male sex was strongly associated with high Canrisk scores (OR = 6.31; 95% CI: 5.12–7.51). Conclusions: Employment in the industrial sector, combined with sedentary behavior and poor dietary habits, is independently associated with higher predicted T2D risk. Workplace prevention strategies should prioritize multicomponent interventions targeting modifiable risk factors, especially in high-risk subgroups such as older, less-educated, and inactive workers.

## 1. Introduction

Type 2 diabetes mellitus (T2D) represents a major global public health challenge, characterized by chronic hyperglycemia, insulin resistance, and progressive β-cell dysfunction [1]. As of 2021, approximately 537 million adults worldwide are affected, a figure projected to rise to nearly 783 million by 2045 [2]. T2D accounts for over 95% of all diabetes cases and is a leading cause of morbidity and mortality. Its growing prevalence entails serious health and socioeconomic consequences, including increased risk of cardiovascular disease, renal failure, neuropathy, retinopathy, and premature death [2].

In Spain, the annual direct healthcare cost of diabetes exceeds €5.1 billion, with additional indirect costs—such as lost productivity—further amplifying the national burden. The estimated prevalence of T2D among Spanish adults aged 20–79 is 14.8%, highlighting the dual challenge of healthcare pressure and economic strain [2,3].

The etiology of T2D is multifactorial. Established risk factors include aging, obesity, sedentary lifestyle, unhealthy dietary habits, and genetic predisposition [4]. In addition, social determinants—such as education, income, and occupational environment—play a crucial role in influencing diabetes risk across diverse populations [5]. The term “diabesity” reflects the strong interrelationship between obesity and T2D, with excess body fat accounting for 60–80% of cases in European populations [4,6].

Due to the chronic and largely preventable nature of T2D, several risk stratification tools have been developed to identify high-risk individuals. These models commonly incorporate age, BMI, waist circumference, physical activity, dietary habits, smoking status, and family history [7]. Recently, lifestyle-integrated risk scores have shown strong predictive validity and practicality in both clinical and community settings [7,8]. For example, one validated score demonstrated predictive accuracy for five-year incident T2D comparable to more complex models [8].

The workplace constitutes a critical yet underexplored environment for T2D prevention. In addition to influencing schedules and routines, the work setting shapes stress levels, physical activity, dietary patterns, and socio-occupational status—all important contributors to metabolic health [9]. Meta-analyses have demonstrated the effectiveness of multicomponent workplace interventions—combining education, exercise, and dietary support—in reducing BMI, glucose levels, and HbA1c [10,11]. A recent umbrella review concluded that such multifaceted programs outperform single-component approaches10. For example, a workplace lifestyle intervention for employees with prediabetes led to short-term improvements in cardiovascular risk factors [12].

Different occupational sectors may contribute to T2D risk in distinct ways. Industrial workers are frequently exposed to high job strain, irregular shift work, long hours, and potential pollutants—all factors linked to elevated T2D risk [13,14]. Rotating and night shifts disrupt circadian rhythms, impair glucose metabolism, and are associated with increased T2D incidence; although moderate-to-vigorous physical activity may partially mitigate these effects [15]. Long working hours, particularly among low socioeconomic groups, have also been associated with increased T2D risk and reduced sleep quality [16].

By contrast, workers in the commerce or service sectors often experience more stable schedules and different socioeconomic characteristics, but may be exposed to prolonged sitting, stress from customer interaction, and irregular eating patterns. Despite these factors, few studies have directly compared T2D risk profiles between commerce and industrial workers using multiple validated tools.

Existing evidence suggests that industrial workers—particularly in male-dominated settings—tend to exhibit higher rates of obesity, increased waist circumference, metabolic syndrome, and T2D risk [17]. However, prior studies frequently lack stratification by sex, socioeconomic status, or key lifestyle factors such as diet and exercise—limiting their utility for tailored prevention strategies.

Recent advances in precision prevention, including the use of polygenic risk scores (PRS) and machine learning models, offer promising methods for improving individual-level risk stratification [18,19,20,21]. Nevertheless, their application in occupational health remains limited.

Understanding how sociodemographic and lifestyle factors intersect is essential. Lower educational attainment and socioeconomic disadvantage increase T2D risk independently of behavioral risk factor [5]. Work environments that promote physical inactivity, long commutes, or limited autonomy may further exacerbate these disparities [22]. Additionally, environmental exposures—such as dust or chemicals in industrial settings—may indirectly contribute to insulin resistance and hyperglycemia [23].

Effective workplace interventions must therefore move beyond educational campaigns. Programs that integrate group-based exercise, dietary counseling, behavior change strategies, stress management, and environmental modifications (e.g., healthy canteens, ergonomic spaces) have demonstrated greater effectiveness [9]. The Mediterranean diet—rich in vegetables, whole grains, unsaturated fats, and legumes—has been linked to an 82% reduction in T2D incidence when combined with physical activity, healthy weight maintenance, and non-smoking behaviors [24].

Despite its relevance, there remains a lack of comparative data on T2D risk and its correlates across occupational sectors in Spain. This study addresses that gap by analyzing a large cohort of over 69,000 Spanish workers using four validated T2D risk scores (QDscore, Finrisk, Canrisk, and TRAQ-D). Risk estimates are stratified by occupational sector (commerce vs. industry), sex, age, education, physical activity, dietary adherence (Mediterranean diet), and smoking status.

The rationale for selecting these two sectors lies in their contrasting physical demands, work schedules, and socioeconomic profiles. Commerce typically involves retail and administrative roles with daytime hours and variable physical demands, whereas industry often entails shift work, physical strain, and lower job autonomy.

Additionally, stratification by sex and educational attainment offers insights into intersecting vulnerabilities. Men generally show higher T2D risk, while women in industrial roles may be especially vulnerable due to combined stress and low autonomy [25]. Education is linked not only to income but also to health literacy, access to nutritious food, and the ability to adopt preventive health behaviors [5,24].

By comparing both mean scores and high-risk prevalence across four instruments, this study aims to clarify how occupational setting and lifestyle interact to shape T2D risk. Multinomial logistic regression models are used to estimate the independent associations of sector, sex, age, education, physical activity, diet quality, and smoking status with elevated risk categories—thus aligning with recommendations for multivariable prediction models [7].

These findings will inform workplace health strategies and policies by identifying high-risk subgroups in need of tailored interventions. In particular, industrial workers with low physical activity and poor dietary patterns may benefit most from preventive workplace programs.

In summary, this study integrates recent evidence—from global epidemiology, occupational health, lifestyle interventions, precision prevention, and socioeconomic determinants—to address a critical gap: sector-stratified, lifestyle-adjusted T2D risk in a large working population. Given the high burden of T2D, the modifiable nature of many risk factors, and the potential for workplace interventions, such data are essential for designing effective, context-sensitive prevention programmes.

These two sectors were selected due to their contrasting working conditions: commerce jobs are generally less physically demanding and more socially oriented, while industrial jobs are associated with greater physical strain, shift work, and lower socioeconomic status—factors hypothesized to influence T2D risk.

We hypothesized that workers in the industrial sector, due to their occupational and socioeconomic characteristics, would present significantly higher T2D risk scores compared to those in the commerce sector.

## 2. Methods

### 2.1. Study Design and Population

**Figure 1:**
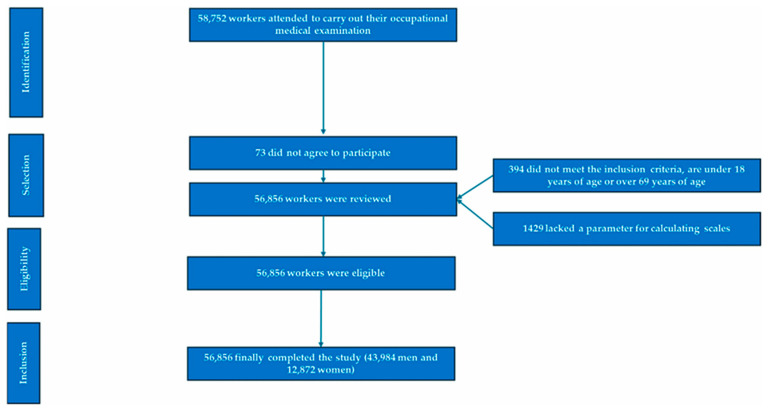
PRISMA diagram illustrating the participant selection process for this study.

This was a cross-sectional analysis of adult employees aged 18 to 69 years, working in the commerce and industry sectors, recruited across Spain between January and December 2023. The sampling frame consisted of randomly selected companies from occupational health records in both sectors. All employees attending routine occupational health assessments during the study period were invited to participate (see Figure 1).

No a priori sample size calculation was performed, as the analysis was based on secondary data from a large occupational health registry. Nonetheless, the final sample of over 69,000 participants ensured sufficient statistical power to detect meaningful differences across sociodemographic and lifestyle strata.

In Spain, all employed individuals have access to universal healthcare services, including regular occupational and primary care. As such, the study population had consistent access to medical follow-up and standardized data collection protocols.

### 2.2. Inclusion and Exclusion Criteria

Inclusion criteria were: age between 18 and 69 years; current full-time or part-time employment in either the commerce or industry sector; ability to read and complete questionnaires in Spanish or Catalan; and provision of written informed consent.

Exclusion criteria included: previously diagnosed diabetes mellitus (type 1 or type 2); pregnancy; inability to complete self-report instruments due to cognitive or language barriers; and acute illness or injury that interfered with participation.

### 2.3. Data Collection

Participants completed a structured questionnaire during their occupational health visit, which gathered sociodemographic data (age, sex, education level), lifestyle behaviors (smoking status, physical activity, diet), and anthropometric measurements. Body weight (±0.1 kg), height (±0.1 cm), and waist circumference were measured following standardized ISAK (International Society for the Advancement of Kinanthropometry) protocols.

#### 2.3.1. Blood Pressure Measurement

Blood pressure was measured using an OMRON-M3 automatic sphygmomanometer (OMRON, Osaka, Japan). Measurements were obtained with the participant seated after at least 10 min of rest. Three readings were taken at one-minute intervals, and the mean of the three was used as the final value

#### 2.3.2. Blood Sample Collection and Processing

Venous blood samples were collected after a minimum fasting period of 12 h using 8.5 mL BD SST II Vacutainer® serum tubes with gel separators (BD reference 366468) (Becton Dickinson, Madrid, Spain). Samples were transported to the laboratory under refrigerated conditions (5 °C to 10 °C) and centrifuged within two hours of collection. Biochemical analyses were performed immediately using an automated clinical chemistry analyzer. LDL cholesterol was estimated using the Friedewald formula unless triglyceride levels were ≥400 mg/dL, in which case direct LDL-C measurement was used. All values were expressed in mg/dL.

#### 2.3.3. Physical Activity Assessment (IPAQ-SF)

Physical activity was assessed using the short form of the International Physical Activity Questionnaire (IPAQ-SF), which evaluates weekly activity across four domains: vigorous activity, moderate activity, walking, and sitting time. Total physical activity was expressed in metabolic equivalent (MET) minutes per week. Participants were classified as “active” if they accumulated ≥600 MET-minutes/week, according to IPAQ scoring protocols [26].

The IPAQ-SF has demonstrated moderate validity against accelerometry (r ≈ 0.30–0.40) and is widely used in epidemiological studies of working-age adults [27,28]. While it tends to overestimate activity levels, its reproducibility and construct validity support its use in large-scale occupational studies [27,29].

#### 2.3.4. Mediterranean Diet Adherence (PREDIMED-MEDAS)

Dietary quality was assessed using the validated 14-item Mediterranean Diet Adherence Screener (MEDAS), developed in the PREDIMED trial [30]. Each affirmative response scores one point, yielding a total score from 0 to 14. A score ≥9 was classified as high adherence. The MEDAS has demonstrated moderate-to-good validity and test–retest reliability (ICC ≈ 0.51–0.61) compared to full food frequency questionnaires [30,31]. Higher MEDAS scores have been associated with lower fasting glucose and triglyceride levels, supporting its construct validity [30].

It should be noted that the MEDAS measures dietary quality, not quantity, and does not capture caloric intake or macronutrient distribution. Therefore, our analysis focused on qualitative dietary adherence rather than energy consumption.

#### 2.3.5. Validated Type 2 Diabetes Risk Scales

Four validated risk scores were used to assess the probability of future T2D development:

- Finrisk: a Finnish score that incorporates age, diet, physical activity, and waist circumference [32,33];
- QDscore: a UK-based model that includes BMI, age, smoking status, and family history [34];
- Canrisk: a Canadian model including age, waist circumference, ethnicity, and behavioral factors [35,36];
- TRAQ-D: a Spanish occupational health tool developed specifically for the working population in Spain [37].

These scores are intended for risk prediction, not diagnosis. Their use allows for the identification of individuals at elevated risk for developing T2D, facilitating timely preventive interventions. This approach aligns with current recommendations emphasizing early detection and primary prevention in occupational health contexts [7,8].

### 2.4. Statistical Analysis

Descriptive statistics were reported as means and standard deviations for continuous variables and as frequencies and percentages for categorical variables. Comparisons between sectors and lifestyle categories were performed using Student’s *t*-tests for continuous variables and Chi-square tests for categorical variables.

Multinomial logistic regression was used to examine independent associations between sex, age group (<40, 40–59, ≥60 years), educational level (primary, secondary, tertiary), occupational sector, smoking status, physical activity (active vs. inactive per IPAQ), and dietary adherence (high ≥ 9 vs. low < 9 per MEDAS) with high-risk categories on each T2D risk score (QDscore, Finrisk, Canrisk, TRAQ-D). Odds ratios (ORs) with 95% confidence intervals (CIs) were calculated.

All analyses were conducted using SPSS version 29.0 (IBM Corp., New York, NY, USA). A two-sided *p*-value of <0.05 was considered statistically significant.

## 3. Results

Table 1 presents the anthropometric, clinical, and lifestyle characteristics of participants across commerce and industry sectors, stratified by sex.

Among women, those employed in the industrial sector were significantly older, had higher BMI and waist circumference, and exhibited more adverse lipid profiles compared to their counterparts in commerce (*p* < 0.001 for all variables). Differences among men were less pronounced but included significantly higher systolic blood pressure and less favorable lipid parameters in the industrial group.

Educational attainment and physical activity levels also differed markedly by sector. In the industrial sector, both men and women showed lower levels of education and higher rates of physical inactivity. Smoking prevalence was higher among men in both sectors, while sectoral differences among women were smaller.

These findings underscore sociodemographic and behavioral disparities between work sectors that may influence metabolic and diabetes risk.

Table 2 displays mean values of the four validated T2D risk scores—QDscore, Finrisk, Canrisk, and TRAQ-D—by sex, sector, and key demographic and lifestyle variables.

Across both sexes and sectors, all risk scores increased with age. Physically inactive individuals and those with low adherence to the Mediterranean diet consistently demonstrated higher mean scores. Notably, participants in the industrial sector—particularly older adults and those with lower education—had significantly elevated mean values for all four risk scores.

These patterns highlight the cumulative impact of modifiable lifestyle factors and occupational environment on predicted diabetes risk.

Table 3 reports the prevalence (%) of participants classified into high-risk categories for each diabetes score across demographic and behavioral strata.

The highest prevalence of elevated T2D risk was observed among older adults (60–69 years), individuals with low educational attainment, physically inactive participants, and those with poor dietary adherence. Industrial sector workers—especially women—demonstrated consistently higher prevalence of high Canrisk and Finrisk scores compared to their commerce counterparts.

These results emphasize the importance of sector-specific preventive approaches targeting high-risk subgroups, particularly in aging and sedentary populations.

Table 4 summarizes the results of multinomial logistic regression analyses assessing independent associations between sociodemographic and lifestyle variables and high-risk classifications across the four T2D risk scores.

Physical inactivity and low Mediterranean diet adherence emerged as the strongest independent predictors of high T2D risk across all models. For instance, physically inactive individuals had up to 12.5 times higher odds of elevated QDscore risk, while poor diet adherence was associated with odds ratios up to 6.6.

Employment in the industrial sector was also significantly associated with increased risk, with odds ratios ranging from 1.23 to 1.98 across different scores. Male sex was a particularly strong predictor of high Canrisk values (OR = 6.31; 95% CI: 5.12–7.51).

These findings provide robust evidence that both individual lifestyle behaviors and broader occupational contexts contribute independently to elevated T2D risk.

## 4. Discussion

In this large cross-sectional study of more than 69,000 working adults in Spain, we identified substantial sector-based differences in predicted type 2 diabetes (T2D) risk using four validated risk scores. Workers in the industrial sector exhibited significantly higher risk profiles than those in the commerce sector, with physical inactivity and poor dietary adherence emerging as the strongest independent predictors of elevated risk. These findings support the multifactorial nature of T2D and the relevance of occupational context in shaping metabolic health outcomes [3,16,31].

### 4.1. Sectoral Differences and Occupational Determinants

Our findings reinforce prior evidence that industrial work environments are associated with greater metabolic risk than service-oriented occupations [38]. Industrial workers often face job strain, shift work, extended hours, physical hazards, and lower job autonomy—factors known to elevate T2D risk. A cohort study from China found that blue-collar men had a 23% higher incidence of T2D compared to white-collar workers, independent of BMI [39]. In our population, industrial workers—especially women—frequently demonstrated co-occurrence of physical inactivity and poor dietary adherence, illustrating how workplace conditions may promote clustering of adverse health behaviors [40].

Although shift work was not explicitly measured, the elevated T2D risk among industrial workers likely reflects circadian rhythm disruption and metabolic dysregulation typical of rotating or night shifts [41,42]. Future studies should incorporate detailed occupational variables such as shift timing, job strain, and control to refine risk estimation [43,44].

### 4.2. Lifestyle Behaviors: Physical Activity and Diet

Consistent with the literature, physical inactivity and low adherence to the Mediterranean diet were independently associated with markedly higher T2D risk scores [45,46,47]. Randomized controlled trials have shown that ≥150 min/week of moderate physical activity can reduce diabetes risk by ~30%, while Mediterranean dietary patterns offer additional benefits [46,48,49].

In our sample, physically active individuals had 35–60% lower odds of high-risk classification across all indices, while high dietary adherence reduced risk by 30–55%. Although the MEDAS score does not quantify caloric intake, its strong associations with metabolic outcomes and reproducibility support its utility for population-level assessment [30,31].

Notably, only a modest correlation was observed between physical activity and diet quality, suggesting that these behaviors operate independently and may require distinct intervention strategies. Cluster-randomized trials have shown synergistic benefits when combining dietary and exercise interventions [45,50].

### 4.3. Sociodemographic Influences: Sex, Age, Education

In line with previous research, male sex and older age were strong independent predictors of elevated T2D risk [41,42,51,52]. Men had particularly high odds of exceeding Canrisk thresholds, consistent with known sex-based physiological and behavioral differences. Age-related β-cell dysfunction, increased adiposity, and insulin resistance contribute to the progressive nature of T2D in aging populations.

Educational attainment also emerged as a significant factor. Participants with lower education had 15–40% higher odds of high-risk scores after adjusting for lifestyle variables. This finding aligns with meta-analytic evidence that low education is a key social determinant of health that operates independently of behaviors [5,53,54]. While our classification did not distinguish between general and health-specific education, the findings highlight the importance of promoting health literacy in lower-education groups.

### 4.4. Comparison with Other Risk Tools and Populations

The use of four validated T2D risk scores enhanced the robustness and generalizability of our findings. Scores developed in non-Spanish populations (QDscore, Finrisk, Canrisk) yielded comparable results to TRAQ-D, a tool designed for occupational cohorts in Spain [37,55]. This concordance suggests that these instruments retain discriminatory validity across populations and settings.

Similar patterns have been observed in other European studies. For example, a German workplace survey found 25% higher Finrisk scores among blue-collar versus office workers, while UK data showed a 28% increased risk among craft and plant operatives [56,57]. Our results thus reflect broader occupational trends within the European context.

Although inclusion of individuals with known diabetes could have provided insight into disease management, we intentionally excluded them to avoid reverse causation and ensure that lifestyle factors represented pre-disease conditions.

Comparing people with and without diabetes might also yield relevant insights. However, we intentionally excluded individuals with diagnosed T2Dto avoid reverse causation and ensure that lifestyle factors reflected predisease status.

### 4.5. Policy Implications and Workplace Prevention

Our findings strongly support the implementation of multicomponent workplace interventions tailored to high-risk occupational settings. Industrial environments, in particular, should prioritize structured physical activity breaks, dietary support programs, and stress reduction strategies. Evidence from recent reviews suggests that such interventions can yield meaningful reductions in body weight (~2.5 kg) and fasting glucose (~5 mg/dL) within 6–12 months [58].

While financial incentives may increase participation, workplace culture, peer support, and managerial involvement are more influential in driving sustainable behavior change [59,60,61]. Industrial settings often present additional barriers—e.g., shift rotations, noise, and limited healthy food options—which require structural modifications such as ergonomic spaces and improved food access [62].

Regular screening using validated tools (e.g., TRAQ-D or Finrisk) can help occupational health services identify high-risk individuals and target resources effectively. A Dutch trial showed that quarterly screening combined with coaching led to a 35% reduction in prediabetes over two years [63]. Such models may be especially relevant in Southern Europe, where adherence to Mediterranean dietary principles may enhance intervention efficacy.

### 4.6. Strengths and Limitations

This study’s strengths include its large sample size, sex-stratified analyses, the use of four validated risk tools, and standardized assessments of physical activity and diet. By adjusting for sociodemographic and behavioral variables, we were able to identify independent contributors to T2D risk.

Limitations include the cross-sectional design, which precludes causal inference. Self-reported measures (IPAQ and MEDAS) are subject to recall and social desirability bias, although both instruments have demonstrated acceptable validity in working populations [27,64]. We lacked data on shift work, income, medication use, and family history, which could confound associations between occupational sector and T2D risk.

Moreover, the study focused on risk prediction rather than clinical diagnosis. Although fasting glucose levels were available, we did not use clinical thresholds or biomarkers such as HbA1c or OGTT for classification. Future research should incorporate these clinical endpoints to improve diagnostic accuracy.

### 4.7. Future Research

Longitudinal follow-up is needed to determine whether elevated risk scores predict incident T2D and to evaluate the impact of workplace interventions on long-term outcomes. Integration of objective measures (e.g., accelerometry, dietary records, biomarkers) would enhance precision [65,66].

The inclusion of polygenic risk scores alongside lifestyle variables may refine stratification and identify individuals most responsive to intervention [67]. Qualitative research exploring barriers to behavior change in industrial settings and cost-effectiveness analyses comparing prevention versus treatment approaches will also be critical for shaping occupational health policy.

## 5. Conclusions

This large cross-sectional study provides robust evidence that employment in the industrial sector—when combined with physical inactivity and low adherence to the Mediterranean diet—is significantly associated with elevated type 2 diabetes (T2D) risk scores among working adults in Spain. These associations remained significant after adjusting for age, sex, education level, and smoking status, underscoring the independent contributions of occupational context and modifiable lifestyle behaviors to metabolic risk.

The observed clustering of adverse health behaviors among industrial workers highlights the importance of considering occupational determinants in the design of prevention strategies. Sector-specific interventions should prioritize physical activity promotion, dietary support, and structural workplace modifications, particularly in high-risk subgroups such as older, less-educated, and sedentary individuals.

The integration of validated T2D risk scores into routine occupational health assessments may facilitate early identification of at-risk workers and support the implementation of targeted, multicomponent preventive programs. These findings contribute to the growing evidence base supporting workplace-centered approaches to the primordial prevention of T2D and emphasize the workplace as a critical setting for improving cardiometabolic health.

## Figures and Tables

**Table 1:** Baseline Characteristics of Participants by Sector and Sex.

	Men			Women		
	Commerce n = 18160	Industry n = 25824		Commerce n = 9288	Industry n = 3584	
	Mean (SD)	Mean (SD)	*p*-Value	Mean (SD)	Mean (SD)	*p*-Value
Age (years)	39.5 (9.8)	39.4 (10.5)	0.225	35.9 (10.1)	41.6 (10.5)	<0.001
Height (cm)	175.0 (6.7)	173.9 (7.0)	<0.001	162.0 (6.4)	160.9 (6.5)	<0.001
Weight (cm)	81.5 (12.5)	81.3 (14.2)	0.064	65.3 (13.4)	68.8 (14.0)	<0.001
Hip	87.5 (8.8)	87.7 (9.0)	0.121	73.7 (7.5)	75.1 (8.0)	<0.001
Cadera (cm)	100.6 (7.9)	99.6 (8.4)	<0.001	97.0 (8.9)	98.1 (9.4)	<0.001
SBP (mmHg)	122.6 (14.4)	124.5 (5.0)	0.024	112.6 (14.2)	117.9 (16.2)	<0.001
DBP (mmHg)	74.5 (10.2)	75.6 (10.5)	0.170	68.9 (9.8)	71.5 (10.7)	<0.001
Total Cholesterol (mg/dL)	193.9 (37.4)	197.5 (38.6)	<0.001	189.4 (35.4)	201.1 (39.3)	<0.001
HDL-cholesterol (mg/dL)	51.1 (6.7)	51.4 (7.0)	<0.001	54.5 (7.9)	52.3 (7.5)	<0.001
LDL-cholesterol (mg/dL)	119.4 (37.7)	121.9 (37.2)	<0.001	117.7 (35.6)	130.6 (38.8)	<0.001
Triglycerides (mg/dL)	119.3 (81.3)	122.4 (84.6)	<0.001	85.4 (37.6)	90.8 (45.8)	<0.001
Glucose (mg/dL)	86.3 (11.9)	88.7 (12.9)	<0.001	84.2 (10.6)	84.3 (11.9)	0.210
	(%)	(%)	*p*-value	(%)	(%)	*p*-value
18–29 years	17.7	20.3	<0.001	32.1	16.5	<0.001
30–39 years	31.8	31.7		32.6	26.9	
40–49 years	33.6	28.5		23.6	31.0	
50–59 years	14.7	16.7		10.3	23.4	
60–69 years	2.2	2.8		1.4	2.2	
Elementary	52.4	36.7	<0.001	90.1	83.7	<0.001
High school	47.6	63.3		9.9	16.3	
Non Physical activity	51.5	55.4	<0.001	42.7	59.4	<0.001
Yes Physical activity	48.5	44.6		57.7	40.6	
Non Mediterranean diet	56.1	59.8	<0.001	44.4	59.8	<0.001
Yes Mediterranean diet	43.9	40.2		55.6	40.2	
Non smokers	70.5	63.0	<0.001	68.0	67.2	0.181
Smokers	29.5	37.0		32.0	32.8	

SBP Systolic blood pressure. DBP Diastolic blood pressure. HDL High density lipoprotein. LDL Low density lipoprotein.

**Table 2:** Mean T2D Risk Scores by Sociodemographic and Lifestyle Variables.

		QD-Score RR *				Finrisk *				Canrisk *				TRAQ-D *		
		Commerce		Industry		Commerce		Industry		Commerce		Industry		Commerce		Industry
Men	n	Mean (SD)	n	Mean (SD)	n	Mean (SD)	n	Mean (SD)	n	Mean (SD)	n	Mean (SD)	n	Mean (SD)	n	Mean (SD)
18–29 years	3224	0.8 (1.2)	5248	1.0 (1.4)	3224	2.2 (3.1)	5248	2.2 (3.1)	3224	14.6 (5.8)	5248	15.1 (5.7)	3224	3.4 (2.1)	5248	3.6 (2.1)
30–39 years	5768	1.0 (1.2)	8184	1.3 (1.7)	5768	3.1 (3.5)	8184	3.9 (3.9)	5768	15.0 (6.6)	8184	17.8 (7.0)	5768	4.6 (2.4)	8184	5.1 (2.6)
40–49 years	6104	1.2 (1.2)	7360	1.5 (1.6)	6104	5.1 (3.8)	7360	6.1 (4.3)	6104	19.7 (7.9)	7360	23.3 (8.8)	6104	5.8 (2.4)	7360	6.2 (2.9)
50–59 years	2664	1.5 (1.2)	4312	1.6 (1.2)	2664	7.9 (4.2)	4312	8.1 (4.3)	2664	28.4 (8.8)	4312	30.1 (8.2)	2664	7.8 (3.2)	4312	8.1 (3.0)
60–69 years	400	1.6 (1.1)	720	1.7 (1.3)	400	9.5 (3.8)	720	9.6 (4.7)	400	35.2 (7.2)	720	36.0 (8.8)	400	10.6 (2.9)	720	10.7 (3.0)
Elementary	9512	1.2 (1.3)	9480	1.3 (1.5)	9512	4.6 (4.1)	9480	5.1 (4.5)	9512	20.2 (8.8)	9480	22.0 (9.5)	9512	5.5 (2.9)	9480	5.9 (3.1)
High school	8648	1.1 (1.1)	16,344	1.4 (1.6)	8648	4.4 (4.2)	16,344	5.0 (4.4)	8648	17.6 (9.0)	16,344	21.1 (9.4)	8648	5.3 (3.0)	16,344	5.6 (3.1)
Non PhA	9344	1.7 (1.4)	14,304	1.9 (1.8)	9344	7.5 (3.6)	14,304	7.9 (3.8)	9344	24.2 (8.5)	14,304	26.4 (8.9)	9344	6.6 (3.1)	14,304	6.9 (3.2)
Yes PhA	8816	0.5 (0.4)	11,520	0.6 (0.3)	8816	1.3 (1.7)	11,520	1.5 (2.1)	8816	13.3 (5.3)	11,520	15.1 (5.7)	8816	4.1 (2.1)	11,520	4.3 (2.4)
Non MD	10,184	1.6 (1.4)	15,440	1.8 (1.8)	10,184	7.1 (3.8)	15,440	7.5 (4.0)	10,184	23.5 (8.7)	15,440	25.8 (8.9)	10,184	6.4 (3.1)	15,440	6.7 (3.2)
Yes MD	7976	0.5 (0.5)	10,384	0.6 (0.3)	7976	1.2 (1.7)	10,384	1.4 (2.1)	7976	13.1 (5.2)	10,384	14.8 (5.7)	7976	4.2 (2.1)	10,384	4.3 (2.4)
Non smokers	12,808	1.0 (1.0)	16,280	1.3 (1.5)	12,808	4.4 (4.1)	16,280	5.2 (4.5)	12,808	18.7 (9.0)	16,280	21.8 (9.6)	12,808	4.6 (2.5)	16,280	5.0 (3.0)
Smokers	5352	1.3 (1.5)	9544	1.4 (1.6)	5352	4.6 (4.3)	9544	4.8 (4.4)	5352	19.4 (8.9)	9544	20.6 (9.2)	5352	7.1 (3.0)	9544	7.2 (3.0)
Women	n	Mean (SD)	n	Mean (SD)	n	Mean (SD)	n	Mean (SD)	n	Mean (SD)	n	Mean (SD)	n	Mean (SD)	n	Mean (SD)
18–29 years	2984	0.9 (1.2)	592	1.2 (3.8)	2984	2.0 (3.1)	592	2.3 (3.4)	2984	8.3 (5.4)	592	8.5 (5.8)	2984	1.2 (2.2)	592	1.9 (2.7)
30–39 years	3024	1.2 (2.2)	960	1.4 (2.9)	3024	2.5 (3.4)	960	3.9 (4.1)	3024	9.1 (6.2)	960	11.2 (7.2)	3024	2.1 (2.5)	960	3.0 (3.2)
40–49 years	2192	1.4 (1.8)	1112	1.6 (1.9)	2192	4.3 (4.0)	1112	5.6 (4.3)	2192	13.8 (7.7)	1112	17.0 (8.7)	2192	3.6 (3.1)	1112	3.9 (2.8)
50–59 years	960	1.6 (1.7)	840	1.7 (1.3)	960	7.6 (4.8)	840	7.9 (3.9)	960	23.1 (9.7)	840	24.1 (8.8)	960	5.4 (3.7)	840	5.6 (3.2)
60–69 years	128	1.7 (1.8)	80	1.8 (1.2)	128	7.8 (2.8)	80	9.4 (4.0)	128	25.9 (5.6)	80	31.1 (7.0)	128	7.1 (2.4)	80	8.6 (5.0)
Elementary	8368	1.4 (2.6)	3000	1.7 (2.1)	8368	3.4 (4.1)	3000	5.5 (4.5)	8368	11.9 (8.3)	3000	17.2 (10.0)	8368	2.6 (3.1)	3000	4.0 (3.4)
High school	920	1.1 (1.7)	584	1.4 (1.8)	920	3.2 (3.6)	584	3.8 (4.0)	920	9.7 (8.2)	584	9.9 (6.7)	920	2.4 (2.6)	584	2.9 (3.1)
Non PhA	3928	2.4 (3.5)	2128	2.5 (2.4)	3928	7.0 (3.8)	2128	7.9 (3.7)	3928	18.0 (8.6)	2128	21.1 (9.2)	3928	3.9 (3.8)	2128	4.9 (3.8)
Yes PhA	5360	0.5 (0.2)	1456	0.6 (0.3)	5360	0.8 (1.4)	1456	1.3 (1.5)	5360	7.0 (3.8)	1456	8.6 (4.9)	5360	1.7 (1.9)	1456	2.2 (2.0)
Non MD	4120	2.3 (3.4)	2144	2.4 (2.4)	4120	6.6 (4.0)	2144	7.8 (3.9)	4120	17.4 (8.7)	2144	20.9 (9.3)	4120	3.8 (3.7)	2144	4.8 (3.7)
Yes MD	5168	0.5 (0.3)	1440	0.6 (0.3)	5168	0.9 (1.6)	1440	1.4 (1.7)	5168	7.1 (4.0)	1440	8.7 (5.0)	5168	1.7 (2.0)	1440	2.2 (2.0)
Non smokers	6320	1.2 (1.8)	2408	1.7 (2.0)	6320	3.1 (3.8)	2408	3.9 (3.7)	6320	10.7 (7.2)	2408	12.7 (7.7)	6320	1.9 (2.8)	2408	3.4 (3.6)
Smokersc	2968	1.5 (3.5)	1176	1.8 (2.2)	2968	3.6 (4.1)	1176	5.9 (4.6)	2968	12.1 (8.8)	1176	17.7 (10.4)	2968	4.1 (3.1)	1176	4.7 (2.7)

SD Standard deviation.. RR Relative risk. TRAQ-D Trinidad Risk Assessment Questionnaire for Type 2 Diabetes Mellitus. PhA Physical activity. (*) Statistical significance in all cases.

**Table 3:** Prevalence of High-Risk Values in T2D Scores by Variables.

		Qd-Score RR > 3 *				Finrisk High *				Canrisk High *				Traq-D High *		
		Commerce		Industry		Commerce		Industry		Commerce		Industry		Commerce		Industry
Men	n	%	n	%	n	%	n	%	n	%	n	%	n	%	n	%
18–29 years	3224	1.9	5248	6.8	3224	0.5	5248	0.6	3224	1.0	5248	1.2	3224	0.7	5248	0.9
30–39 years	5768	6.0	8184	9.6	5768	1.1	8184	1.4	5768	2.1	8184	3.3	5768	1.1	8184	1.3
40–49 years	6104	6.6	7360	11.3	6104	1.4	7360	4.1	6104	7.2	7360	12.8	6104	1.5	7360	1.6
50–59 years	2664	12.3	4312	12.7	2664	4.1	4312	6.7	2664	27.9	4312	31.2	2664	1.7	4312	2.0
60–69 years	400	12.9	720	13.4	400	12.0	720	12.4	400	62.0	720	63.9	400	1.8	720	2.2
Elementary	9512	7.3	9480	8.4	9512	2.5	9480	3.2	9512	9.6	9480	12.2	9512	2.2	9480	2.3
High school	8648	6.4	16,344	10.8	8648	1.8	16,344	3.0	8648	7.8	16,344	11.3	8648	1.8	16,344	1.9
Non PhA	9344	12.6	14,304	17.1	9344	4.2	14,304	5.7	9344	16.2	14,304	20.3	9344	3.9	14,304	4.1
Yes PhA	8816	1.3	11,520	1.9	8816	0.8	11,520	1.2	8816	1.2	11,520	1.5	8816	0.8	11,520	0.9
Non MD	10,184	12.2	15,440	16.4	10,184	4.0	15,440	5.3	10,184	15.5	15,440	18.7	10,184	3.7	15,440	3.8
Yes MD	7976	2.2	10,384	2.7	7976	1.1	10,384	1.5	7976	1.9	10,384	2.2	7976	1.1	10,384	1.2
Non smokers	12,808	5.6	16,280	10.0	12,808	2.1	16,280	3.3	12,808	8.5	16,280	10.5	12,808	1.9	16,280	2.1
Smokers	5352	9.9	9544	11.2	5352	2.4	9544	3.5	5352	8.8	9544	12.6	5352	2.4	9544	2.7
Women	n	%	n	%	n	%	n	%	n	%	n	%	n	%	n	%
18–29 years	2984	4.1	592	5.8	2984	0.5	592	0.7	2984	5.4	592	5.9	2984	0.6	592	0.9
30–39 years	3024	9.0	960	11.8	3024	1.1	960	2.1	3024	9.3	960	15.8	3024	1.2	960	1.6
40–49 years	2192	10.2	1112	14.4	2192	5.8	1112	7.5	2192	17.5	1112	26.6	2192	1.8	1112	2.2
50–59 years	960	13.6	840	17.4	960	12.5	840	16.2	960	51.6	840	58.1	960	2.1	840	2.5
60–69 years	128	15.9	80	20.2	128	15.7	80	17.9	128	58.8	80	61.3	128	2.9	80	3.3
Elementary	8368	10.8	3000	15.5	8368	6.6	3000	7.2	8368	15.9	3000	33.1	8368	2.5	3000	2.7
High school	920	7.1	584	13.8	920	4.9	584	6.1	920	13.0	584	21.2	920	1.6	584	1.9
Non PhA	3928	20.7	2128	24.2	3928	6.8	2128	7.2	3928	35.4	2128	47.7	3928	3.0	2128	3.2
Yes PhA	5360	3.9	1456	4.4	5360	0.8	1456	1.1	5360	2.9	1456	3.6	5360	0.2	1456	0.3
Non MD	4120	19.8	2144	22.1	4120	6.5	2144	6.9	4120	33.3	2144	42.8	4120	2.9	2144	3.0
Yes MD	5168	5.1	1440	6.5	5168	1.3	1440	1.6	5168	4.8	1440	6.2	5168	0.4	1440	0.5
Non smokers	6320	10.0	2408	13.2	6320	4.8	2408	5.2	6320	10.9	2408	13.7	6320	1.9	2408	2.2
Smokers	2968	11.2	1176	14.8	2968	5.9	1176	6.6	2968	17.8	1176	28.9	2968	2.1	1176	2.5

RR Relative risk. TRAQ-D Trinidad Risk Assessment Questionnaire for Type 2 Diabetes Mellitus. PhA Physical activity. (*) Statistical significance in all cases.

**Table 4:** Multinomial Logistic Regression of Risk Factors for High T2D Scores.

	QD-Score RR > 3 *	Finrisk High *	Canrisk High *	TRAQ-D High *
	OR (95% CI)	OR (95% CI)	OR (95% CI)	OR (95% CI)
Women	1	1	1	1
Men	0.48 (0.44–0.52)	1.08 (1.05–1.11)	6.31 (5.12–7.51)	1.29 (1.22–1.36)
18–29 years	1	1	1	1
30–39 years	1.20 (1.16–1.24)	1.59 (1.50–1.69)	1.25 (1.20–1.30)	1.39 (1.29–1.49)
40–49 years	1.26 (1.21–1.31)	3.41 (2.90–3.91)	2.18 (1.85–2.31)	2.30 (2.01–2.61)
50–59 years	1.40 (1.33–1.47)	6.16 (5.01–7.32)	4.48 (3.58–5.59)	4.75 (3.96–5.55)
60–69 years	1.56 (1.48–1.65)	10.05 (8.65–11.46)	7.94 (6.50–9.28)	7.33 (6.12–8.43)
Elementary	1	1	1	1
High school	1.39 (1.35–1.44)	1.20 (1.15–1.25)	1.82 (1.72–1.93)	1.18 (1.14–1.22)
Commerce	1	1	1	1
Industry	1.27 (1.22–1.32)	1.23 (1.18–1.24)	1.98 (1.88–2.09)	1.43 (1.37–1.50)
Yes physical activity	1	1	1	1
Non physical activity	12.49 (11.19–13.80)	8.88 (7.87–9.90)	6.91 (6.27–7.55)	5.52 (4.86–6.18)
Yes mediterranean diet	1	1	1	1
Non mediterranean diet	6.62 (5.80–7.45)	4.37 (3.56–5.18)	4.24 (3.82–4.66)	2.82 (2.30–3.35)
Non smokers	1	1	1	1
Smokers	1.17 (1.13–1.21)	1.09 (1.06–1.13)	1.23 (1.18–1.29)	1.38 (1.29–1.48)

OR Odss ratio RR Relative risk. TRAQ-D Trinidad Risk Assessment Questionnaire for Type 2 Diabetes Mellitus (*) Statistical significance in all cases.

## Data Availability

The dataset supporting the findings of this study is stored in a secure and access-restricted database managed by ADEMA University School. The designated Data Protection Officer overseeing this process is Ángel Arturo López González.

## References

[1] Magliano D.J., Boyko E.J. (2021). IDF Diabetes Atlas 10th edition scientific committee. IDF DIABETES ATLAS [Internet].

[2] World Health Organization (2024). Diabetes Fact Sheet [Internet].

[3] GBD 2021 Diabetes Collaborators (2023). Global, regional, and national burden of diabetes from 1990 to 2021, with projections of prevalence to 2050: A systematic analysis for the Global Burden of Disease Study 2021. Lancet.

[4] Malik V.S., Hu F.B. (2022). The role of sugar-sweetened beverages in the global epidemics of obesity and chronic diseases. Nat. Rev. Endocrinol..

[5] Hill-Briggs F., Adler N.E., Berkowitz S.A., Chin M.H., Gary-Webb T.L., Navas-Acien A., Thornton P.L., Haire-Joshu D. (2020). Social Determinants of Health and Diabetes: A Scientific Review. Diabetes Care.

[6] Aguiló Juanola M.C., López-González A.A., Tomás-Gil P., Paublini H., Tárraga-López P.J., Ramírez-Manent J.I. (2024). Influence of tobacco consumption on the values of different cardiometabolic risk scales in 418,343 spanish workers. Acad. J. Health Sci..

[7] Zhang Y., Yang Y., Huang Q., Zhang Q., Li M., Wu Y. (2023). The effectiveness of lifestyle interventions for diabetes remission on patients with type 2 diabetes mellitus: A systematic review and meta-analysis. Worldviews Evid. Based Nurs..

[8] Buss V.H., Varnfield M., Harris M., Barr M. (2021). Validation of a lifestyle-based risk score for type 2 diabetes mellitus in Australian adults. Prev. Med. Rep..

[9] Fitzpatrick-Lewis D., Ali M.U., Horvath S., Nagpal S., Ghanem S., Sherifali D. (2022). Effectiveness of Workplace Interventions to Reduce the Risk for Type 2 Diabetes: A Systematic Review and Meta-Analysis. Can. J. Diabetes.

[10] Rosenfeld R.M., Grega M.L., Karlsen M.C., Dabrh A.M.A., Aurora R.N., Bonnet J.P., Donnell L., Fitzpatrick S.L., Frates B., Joy E.A. (2025). Lifestyle Interventions for Treatment and Remission of Type 2 Diabetes and Prediabetes in Adults: A Clinical Practice Guideline From the American College of Lifestyle Medicine. Am. J. Lifestyle Med..

[11] Brinkmann C., Hof H., Gysan D.B., Albus C., Millentrup S., Bjarnason-Wehrens B., Latsch J., Herold G., Wegscheider K., Heming C. (2023). Lifestyle intervention reduces risk score for cardiovascular mortality in company employees with pre-diabetes or diabetes mellitus—A secondary analysis of the PreFord randomized controlled trial with 3 years of follow-up. Front. Endocrinol..

[12] Loeb T.B., Ramm K., Gholami M., Shedd K., Soetenga S., Bhagat M., Jackson N.J., Chung U.Y.R., Duru O.K., Mangione C.M. (2024). Implementation lessons learned from the University of California’s Diabetes Prevention Program Initiative. BMC Public Health.

[13] Mortensen J., Clark A.J., Lange T., Andersen G.S., Goldberg M., Ramlau-Hansen C.H., Head J., Kivimäki M., Madsen I., Leineweber C. (2018). Informal caregiving as a risk factor for type 2 diabetes in individuals with favourable and unfavourable psychosocial work environments: A longitudinal multi-cohort study. Diabetes Metab..

[14] Viklund A., Andersson T., Selander J., Kader M., Albin M., Bodin T., Härmä M., Ljungman P., Bigert C. (2023). Night and shift work patterns and incidence of type 2 diabetes and hypertension in a prospective cohort study of healthcare employees. Scand. J. Work Environ. Health.

[15] Vézina-Im L.A., Morin C.M., Desroches S. (2021). Sleep, Diet and Physical Activity Among Adults Living with Type 1 and Type 2 Diabetes. Can. J. Diabetes.

[16] Chen W.C., Yang H.Y. (2023). Relationship of long working hours and night shift working hours with incident diabetes: A retrospective cohort study in Taiwan. Ann. Epidemiol..

[17] Busquets-Cortés C., Bennasar-Veny M., López-González A.A., Fresneda S., Aguiló A., Yanez A. (2021). Fatty liver index and progression to type 2 diabetes: A 5-year longitudinal study in Spanish workers with pre-diabetes. BMJ Open..

[18] Liu J., Wang L., Cui X., Shen Q., Wu D., Yang M., Dong Y., Liu Y., Chen H., Yang Z. (2023). Polygenic Risk Score, Lifestyles, and Type 2 Diabetes Risk: A Prospective Chinese Cohort Study. Nutrients.

[19] Mohsen F., Al-Absi H.R.H., Yousri N.A., El Hajj N., Shah Z. (2023). A scoping review of artificial intelligence-based methods for diabetes risk prediction. NPJ Digit. Med..

[20] Al-Absi H.R.H., Pai A., Naeem U., Mohamed F.K., Arya S., Sbeit R.A., Bashir M., El Shafei M.M., El Hajj N., Alam T. (2024). DiaNet v2 deep learning based method for diabetes diagnosis using retinal images. Sci. Rep..

[21] Vicente-Herrero M.T., Egea-Sancho M., Ramírez Iñiguez de la Torre M.V., López-González A.A. (2024). Relación de los índices de adiposidad visceral (VAI) y adiposidad disfuncional (DAI) con las escalas de riesgo de resistencia a la insulina y prediabetes. Acad. J. Health Sci..

[22] Mestre-Font M., Busquets-Cortés C., Ramírez-Manent J.I., Tomás-Gil P., Paublini H., López-González A.A. (2024). Influence of sociodemographic variables and healthy habits on the values of type 2 diabetes risk scales. Acad. J. Health Sci..

[23] Meo S.A., Muneif Y.A.B., BenOmran N.A., AlSadhan M.A., Hashem R.F., Alobaisi A.S. (2020). Prevalence of Pre Diabetes and Type 2 Diabetes Mellitus among cement industry workers. Pak. J. Med. Sci..

[24] Celada Roldán C., López Diez J., Rider F., Tárraga Marcos A., Tárraga Marcos M.L., Tárraga López P.J., Gallegos I.R., Arroyo M.M., Manent J.I.R., González Á.A.L. (2024). Impact of adherence to the Mediterranean diet on health-related quality of life in poorly controlled diabetics. Acad. J. Health Sci..

[25] Benavides F.G., Delclós J., Serra C. (2018). Estado del bienestar y salud pública, una relación que debe ser actualizada. Gac. Sanit..

[26] Mestre Font M., Busquets-Cortés C., Ramírez-Manent J.I., Vallejos D., Sastre Alzamora T., López-González A.A. (2024). Influence of sociodemographic variables and healthy habits on the values of cardiometabolic risk scales in 386924 spanish workers. Acad. J. Health Sci..

[27] Meh K., Jurak G., Sorić M., Rocha P., Sember V. (2021). Validity and Reliability of IPAQ-SF and GPAQ for Assessing Sedentary Behaviour in Adults in the European Union: A Systematic Review and Meta-Analysis. Int. J. Envrion. Res. Public Health.

[28] Murtagh E.M., Murphy M.H., Milton K., Roberts N.W., O’Gorman C.S., Foster C. (2020). Interventions outside the workplace for reducing sedentary behaviour in adults under 60 years of age. Cochrane Database Syst. Rev..

[29] Sember V., Meh K., Sorić M., Starc G., Rocha P., Jurak G. (2020). Validity and Reliability of International Physical Activity Questionnaires for Adults across EU Countries: Systematic Review and Meta Analysis. Int. J. Envrion. Res. Public Health.

[30] Mestre-Font M., Busquets-Cortés C., Ramírez-Manent J.I., Tomás-Gil P., Paublini H., López-González A.A. (2024). Influence of sociodemographic variables and healthy habits on the values of overweight and obesity scales in 386,924 Spanish workers. Acad. J. Health Sci..

[31] Schröder H., Zomeño M.D., Martínez-González M.A., Salas-Salvadó J., Corella D., Vioque J., Romaguera D., Martínez J.A., Tinahones F.J., Miranda J.L. (2021). Validity of the energy-restricted Mediterranean Diet Adherence Screener. Clin. Nutr..

[32] Lindström J., Tuomilehto J. (2003). The diabetes risk score: A practical tool to predict type 2 diabetes risk. Diabetes Care.

[33] Saaristo T., Peltonen M., Lindström J., Saarikoski L., Sundvall J., Eriksson J.G., Tuomilehto J. (2005). Cross-sectional evaluation of the Finnish Diabetes Risk Score: A tool to identify undiagnosed type 2 diabetes, abnormal glucose tolerance and metabolic syndrome. Diabetes Vasc. Dis. Res..

[34] Hippisley-Cox J., Coupland C., Robson J., Sheikh A., Brindle P. (2009). Predicting risk of type 2 diabetes in England and Wales: Prospective derivation and validation of QDScore. BMJ..

[35] Robinson D.J., Coons M., Haensel H., Vallis M., Yale J.F. (2018). Diabetes Canada Position Statement: Screening for type 1 and type 2 diabetes. Can. J. Diabetes.

[36] Bird M., Cerutti S., Jiang Y., Srugo S.A., de Groh M. (2022). Implementation of the CANRISK Tool: A Qualitative Exploration Among Allied Health Professionals in Canada. Can. J. Diabetes.

[37] Latchan Z., Seereeram R., Kamalodeen A., Sanchez S., Deonarine U., Sinanan R., Mungrue K. (2010). TRAQ-D (Trinidad Risk Assessment Questionnaire for Type 2 Diabetes Mellitus): A cheap, reliable, non-invasive screening tool for diabetes. Br. J. Diabetes Vasc. Dis..

[38] Trudel X., Brisson C., Talbot D., Gilbert-Ouimet M., Milot A. (2021). Long Working Hours and Risk of Recurrent Coronary Events. J. Am. Coll. Cardiol..

[39] Camero A., Muriel J.L., Morell N., Lurquin M., López-González A.A., Serra-Capó A., Villaroel G. (2024). Risk of insulin resistance appliying 3 different scales in 703,472 spanish workers: Associated variables. J. Cin. TrialsExp Investig..

[40] Ismail L., Materwala H., Al Kaabi J. (2021). Association of risk factors with type 2 diabetes: A systematic review. Comput. Struct. Biotechnol. J..

[41] Gan Y., Yang C., Tong X., Sun H., Cong Y., Yin X., Li L., Cao S., Dong X., Gong Y. (2015). Shift work and diabetes mellitus: A meta-analysis of observational studies. Occup. Environ. Med..

[42] Martín-Peláez S., Fito M., Castaner O. (2020). Mediterranean Diet Effects on Type 2 Diabetes Prevention, Disease Progression, and Related Mechanisms. A Review. Nutrients.

[43] Touitou Y., Reinberg A., Touitou D. (2017). Association between light at night, melatonin secretion, sleep deprivation, and the internal clock: Health impacts and mechanisms of circadian disruption. Life Sci..

[44] Chandrasekaran P., Weiskirchen R. (2024). The Role of Obesity in Type 2 Diabetes Mellitus-An Overview. Int. J. Mol. Sci..

[45] Xie F., Hu K., Fu R., Zhang Y., Xiao K., Tu J. (2024). Association between night shift work and the risk of type 2 diabetes mellitus: A cohort-based meta-analysis. BMC Endocr. Disord..

[46] Bull F.C., Al-Ansari S.S., Biddle S., Borodulin K., Buman M.P., Cardon G., Carty C., Chaput J.-P., Chastin S., Chou R. (2020). World Health Organization 2020 guidelines on physical activity and sedentary behaviour. Br. J. Sports Med..

[47] Hu M., Li B., Xia J., Yin C., Yang Y. (2024). Causal Relationship between Television Viewing Time, Cardiovascular Diseases, and Potential Mechanisms. Arq. Bras. Cardiol..

[48] D’Ettorre G., Pellicani V., Caroli A., Greco M. (2020). Shift work sleep disorder and job stress in shift nurses: Implications for preventive interventions. Med. Lav..

[49] Aranceta-Bartrina J., Partearroyo T., López-Sobaler A.M., Ortega R.M., Varela-Moreiras G., Serra-Majem L., Pérez-Rodrigo C. (2019). Updating the Food-Based Dietary Guidelines for the Spanish Population: The Spanish Society of Community Nutrition (SENC) Proposal. Nutrients.

[50] Magkos F., Hjorth M.F., Astrup A. (2020). Diet and exercise in the prevention and treatment of type 2 diabetes mellitus. Nat. Rev. Endocrinol..

[51] Bonilla-Escobar B.A., Borrell L.N., Del Cura-González I., Sánchez-Perruca L., Escortell-Mayor E., Franco M. (2020). Type 2 diabetes prevalence among Andean immigrants and natives in a Southern European City. Acta Diabetol..

[52] Umpierrez G.E., Pasquel F.J. (2017). Management of Inpatient Hyperglycemia and Diabetes in Older Adults. Diabetes Care.

[53] Allen L., Williams J., Townsend N., Mikkelsen B., Roberts N., Foster C., Wickramasinghe K. (2017). Socioeconomic status and non-communicable disease behavioural risk factors in low-income and lower-middle-income countries: A systematic review. Lancet Glob. Health.

[54] Hernández-Teixidó C., López-Simarro F., Arranz Martínez E., Escobar Lavado F.J., Miravet Jiménez S. (2023). Vulnerabilidad y determinantes sociales en diabetes. Semergen.

[55] Garcia-Samuelsson M., Tarraga-Lopez P.J., Lopez-González A.A., Busquets-Cortes C., Obrador de Hevia J., Ramirez-Manent J.I. (2025). Evaluation of Type 2 Diabetes Risk in Individuals With or Without Metabolically Healthy Obesity. Biology.

[56] Hung H.H.Y., Chan E.Y.Y., Chow E.Y.K., Chung G.K.K., Lai F.T.T., Yeoh E.K. (2022). Non-skilled occupation as a risk factor of diabetes among working population: A population-based study of community-dwelling adults in Hong Kong. Health Soc. Care Community.

[57] Carlsson S., Andersson T., Talbäck M., Feychting M. (2020). Incidence and prevalence of type 2 diabetes by occupation: Results from all Swedish employees. Diabetologia.

[58] Brand S.L., Thompson Coon J., Fleming L.E., Carroll L., Bethel A., Wyatt K. (2017). Whole-system approaches to improving the health and wellbeing of healthcare workers: A systematic review. PLoS ONE.

[59] Prince S.A., Rasmussen C.L., Biswas A., Holtermann A., Aulakh T., Merucci K., Coenen P. (2021). The effect of leisure time physical activity and sedentary behaviour on the health of workers with different occupational physical activity demands: A systematic review. Int. J. Behav. Nutr. Phys. Act..

[60] Mitchell M.S., Orstad S.L., Biswas A., Oh P.I., Jay M., Pakosh M.T., Faulkner G. (2020). Financial incentives for physical activity in adults: Systematic review and meta-analysis. Br. J. Sports Med..

[61] Siguero M.A., Maqueda J., Marqués F., Sagües M.J., Solé M.D. (2021). Evaluation of the Effectiveness of Workplace Health Promotion Programs from 2000 to 2020: Literature Review. Open J. Prev. Med..

[62] Melnyk B.M., Kelly S.A., Stephens J., Dhakal K., McGovern C., Tucker S., Hoying J., McRae K., Ault S., Spurlock E. (2020). Interventions to Improve Mental Health, Well-Being, Physical Health, and Lifestyle Behaviors in Physicians and Nurses: A Systematic Review. Am. J. Health Promot..

[63] Fernández-Figares Vicioso M.P., Riutord Sbert P., López-González Á.A., Ramírez-Manent J.I., Del Barrio Fernández J.L., Herrero M.T.V. (2025). Risk of Insulin Resistance: Comparison of the Commerce vs. Industry Sector and Associated Variables. Diseases.

[64] Cho S.M.J., Koyama S., Honigberg M.C., Surakka I., Haidermota S., Ganesh S., Patel A.P., Bhattacharya R., Lee H., Kim H.C. (2023). Genetic, sociodemographic, lifestyle, and clinical risk factors of recurrent coronary artery disease events: A population-based cohort study. Eur. Heart J..

[65] Lam B., Catt M., Cassidy S., Bacardit J., Darke P., Butterfield S., Alshabrawy O., Trenell M., Missier P. (2021). Using Wearable Activity Trackers to Predict Type 2 Diabetes: Machine Learning-Based Cross-sectional Study of the UK Biobank Accelerometer Cohort. JMIR Diabetes.

[66] Godevithana J., Wijesinghe C.J., Wijesinghe M.S.D. (2024). Paper-based and mobile application-based self-monitoring tool for healthy dietary intake, development and applicability: A non-randomized trial. BMC Digit Health.

[67] Medina C., Monge A., Denova-Gutiérrez E., López-Ridaura R., Barquera S., Romieu I., Lajous M. (2022). Validity and reliability of the International Physical Activity Questionnaire (IPAQ) long-form in a subsample of female Mexican teachers. Salud Publica Mex..

